# Splenic Embolization to Manage Thrombocytopenia in Cancer Patients: Case Reports and Review of the Literature

**DOI:** 10.1155/crh/3779663

**Published:** 2026-02-13

**Authors:** Zahra Ridha, Kiat Tsong Tan, Michael Connolly, Radhika Yelamanchili, Rachel VanderMeer, Mohammad Refaei

**Affiliations:** ^1^ Niagara Regional Campus, Michael G. DeGroote School of Medicine, McMaster University, Hamilton, Canada, mcmaster.ca; ^2^ Medical Imaging Department, Niagara Health, St. Catharines, Canada; ^3^ Radiology Department, McMaster University, Hamilton, Canada, mcmaster.ca; ^4^ Oncology, McMaster University, Hamilton, Canada, mcmaster.ca; ^5^ DoM Hematology and Thromboembolism, McMaster University, Hamilton, Canada, mcmaster.ca

**Keywords:** cancer, chemotherapy, interventional radiology, partial splenic embolization, thrombocytopenia

## Abstract

Thrombocytopenia is a common complication of cancer and its management. Platelet count guides patients’ cancer treatment, as a safe threshold is required before initiating a chemotherapy regimen. Preventing thrombocytopenia in cancer patients is essential to avoid dose reduction or delay of chemotherapy. Partial splenic embolization (PSE) is a procedure that can increase platelet count in cancer patients, allowing them to initiate/resume chemotherapy and receive other treatments such as surgery and tumor ablation. Herein, we report two cases of cancer patients that underwent a PSE to improve thrombocytopenia in order to receive chemotherapy. One patient had a successful procedure, although they had a recurrence of thrombocytopenia 18 months later requiring repeat PSE. The other patient suffered serious complications as a result of unintentional total embolization of the spleen and the tail of the pancreas, resulting in necrotizing pancreatitis that rendered her unable to start chemotherapy. We analyzed both cases to provide insight on the safety and effectiveness of the procedure for cancer patients.

## 1. Introduction

Thrombocytopenia is a common complication of cancer and its management [1]. This can occur by one or more pathophysiological mechanisms such as myelosuppression, immune activation, increased consumption (e.g., DIC), or sequestration due to splenomegaly [1, 2]. The latter can be related to hepatobiliary cancer or preexisting medical comorbidities such as liver cirrhosis. Platelet count can guide patients’ cancer treatment, as a safe threshold is required before initiating a chemotherapy regimen. Resolving thrombocytopenia of cancer patients is essential to avoid dose reduction or delay of chemotherapy [3]. Reducing the relative dose intensity of chemotherapy lowers response rate and patient survival [4].

Thrombocytopenia can be mitigated by a number of interventions, such as corticosteroids, thrombopoietin‐receptor agonists, and adjustment of chemotherapy dose or interval between cycles [4, 5]. Partial splenic embolization (PSE) is a procedure that can increase platelet count in cancer patients [6]. During a PSE, some of the arterial branches that supply the spleen are occluded with an embolic agent, decreasing arterial blood supply to select regions, leading to infarction [7]. This reduces platelet sequestration in the spleen [8]. The resulting increase in platelet count enables cancer patients to receive their chemotherapy and other treatments such as surgery, tumor ablation, and radioactive injections [9–13].

In the literature, most cancer patients did not experience serious complications after a PSE. Common complications reported were pain and fever [9, 11, 14]. Nausea, pulmonary consolidation/atelectasis, effusion, pneumonia, or ascites occurred in some patients post‐PSE [6, 9, 11, 14, 15]. There are a limited number of cases in which patients faced more serious consequences [16–18]. The hospitalization time post‐PSE varied, though it was typically in the range of 1–6 days, given there were no complications or measures requiring extended hospitalization [6, 11, 14, 15, 19].

This case report reviews two cases of cancer patients with different underlying types of cancer and comorbidities who underwent PSE and had different outcomes and complications. The two patients provided informed consent for the publication of their anonymized clinical information.

## 2. Case 1

A 57‐year‐old patient diagnosed with metastatic colorectal cancer presented with thrombocytopenia (platelet count 65 × 10^9^/L). They had been on first‐line palliative FOLFIRI–bevacizumab chemotherapy treatment as a result of unresectable, worsening lung and liver metastases. They previously had a liver resection to remove bilateral lesions, as well as liver embolization. Splenomegaly (19 cm) in the setting of liver disease and portal hypertension was thought to be a major contributor to their thrombocytopenia, along with chemotherapy‐related myelosuppression. Chemotherapy was delayed repeatedly, and their cancer progressed as a result. Their cancer‐associated portal vein thrombosis could not be treated by anticoagulation due to thrombocytopenia. PSE was performed by interventional radiology after a formal hematology consult. The patient underwent embolization of the lower pole branch of the splenic artery with 300–500‐μm particles until there was partial stasis of blood flow. The performing interventional radiologist estimated that 30%–35% embolization of the middle to lower pole of the spleen was attained. After the procedure, the patient was hospitalized for 2 days with middle to upper abdominal tenderness and pain, managed with hydromorphone. They had nausea and vomiting thought to be due to their analgesia.

**Figure 1:**
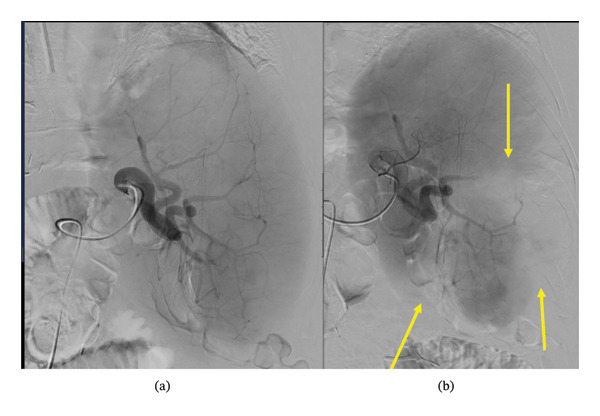
Angiogram images from the second PSE performed on the patient in Case 1. (a) Pre‐embolization digital subtraction angiogram image from the splenic artery demonstrating the arterial branches and parenchymal enhancement. (b) Post‐embolization digital subtraction angiogram image from the splenic artery demonstrating the pruning/occlusion of some of the arterial branches and loss of parenchymal enhancement (yellow arrows).

The PSE increased their platelet count from 65 × 10^9^/L to 150 × 10^9^/L 5 days after PSE and peaked at 174 × 10^9/L 19 days post‐PSE (the target for chemotherapy was over 75 × 10^9^/L). This allowed restarting chemotherapy and thromboprophylaxis a few weeks after the procedure. 18 months later, their thrombocytopenia recurred, precluding chemotherapy. As a result, the patient underwent a second PSE, following a similar technique, embolizing the lower pole splenic vessels to stasis (Figure 1). This time, the performing interventional radiologist estimated that 20 percent of the splenic volume was embolized. Platelet count improved from 73 × 10^9^/L to 117 × 10^9^/L five days after PSE without significant complications, allowing for resumption of chemotherapy.

## 3. Case 2

**Figure 2:**
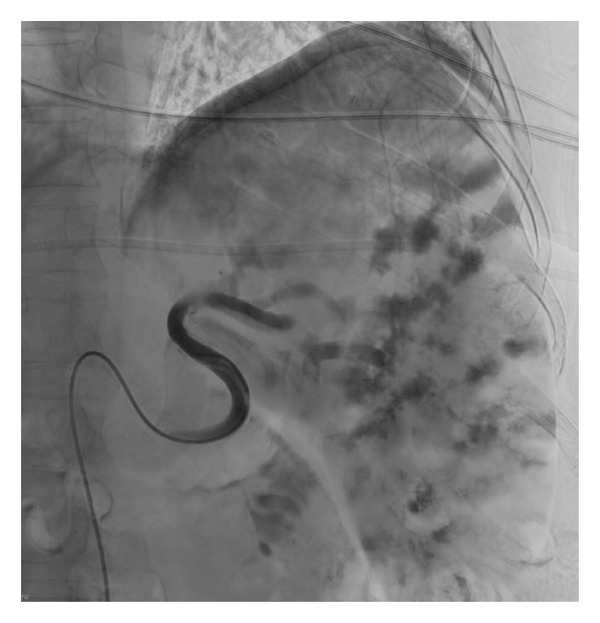
Angiography showing a 5‐Fr catheter in the splenic artery of the patient in Case 2. The lower pole artery was subselectively catheterized using a 2.8‐Fr catheter (not shown).

A 63‐year‐old patient diagnosed with invasive ductal carcinoma (Stage IA HER2‐positive breast cancer) presented with thrombocytopenia. They had a history of colorectal cancer (resected), severe hepatic steatosis, massive splenomegaly (19.5 cm), and psoriatic arthritis. Baseline platelet count was 80–90 × 10^9^/L. The patient underwent a lumpectomy for breast cancer, requiring adjuvant combined chemoradiation therapy within a specific timeframe. A platelet count over 100 × 10^9^/L was set as the target in order to proceed with chemotherapy (paclitaxel and trastuzumab). Thrombocytopenia was likely multifactorial, related to splenomegaly, secondary immune thrombocytopenic purpura, and medication effect (leflunomide). A trial of leflunomide interruption and a taper course of corticosteroids was initiated by her hematologists, without improving her platelet count. As a result, she underwent a PSE (Figure 2). The intention was to only embolize the lower pole splenic artery using 500–700‐μm and 700–900‐μm beads until stasis, such that only a portion of the spleen was infarcted. She did not have any immediate complications.

**Figure 3:**
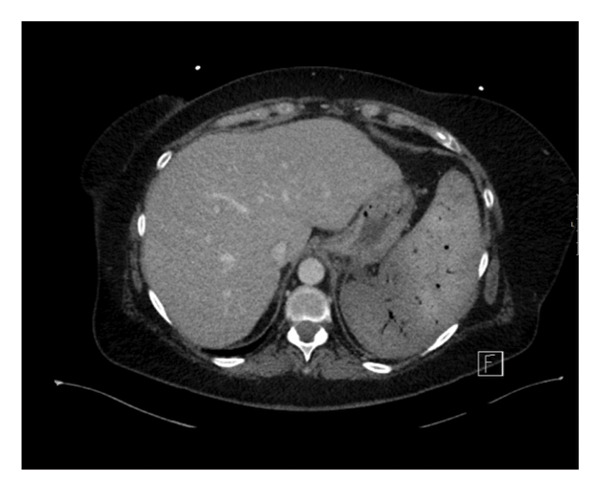
Complete infarction of the spleen on follow‐up CT (axial slice) of Case 2.

However, a week after the procedure, the patient developed severe abdominal pain and was found to have significant splenic infarct and severe necrotizing pancreatitis. This was accompanied by severe hyponatremia, acidosis, and extensive occlusive splenic and portal vein thrombosis post‐PSE. The patient was hospitalized, and management of the pancreatitis was instituted with IV fluids and pain medications, as well as anticoagulation for splanchnic vein thrombosis. Ultimately, CT revealed that the entire spleen was infarcted (Figure 3) as well as the tail of the pancreas. Peri‐splenic and pancreatic fluid collections were visualized, suspicious for abscess. The patient was treated with broad‐spectrum antibiotics. Their clinical course was further complicated by a severe gastrointestinal bleed requiring readmission to the ICU and management under the guidance of a GI interventionalist. Lastly, the patient developed a large left‐sided pleural effusion that was likely linked to the PSE. The left lobe was completely collapsed, requiring abdominal collections and pleural effusions to be drained.

Although the platelet count increased to 873 × 10^9^/L 21 days after the PSE, the patient was no longer a candidate for adjuvant chemotherapy, as they had missed the recommended initiation window and had serious complications.

## 4. Discussion

PSEs can increase the platelet count of thrombocytopenic cancer patients and are usually successful in doing so within a relatively short period after the procedure. In a retrospective study by Kauffman et al., 96.3% of cancer patients (*n* = 27) achieved the desired platelet count of > 150 × 10^9^/L post‐PSE, most of whom reached this value in a mean time of 10 days [14]. A prospective study corroborated similar results; 94% of patients (*n* = 33) met the target platelet count (> 130 × 10^9^/L) after the procedure, with the majority doing so two weeks post‐PSE [11]. Hill et al. demonstrated that the rise in platelet count post‐PSE is durable in cancer patients with hypersplenism‐related thrombocytopenia and continues after chemotherapy is reinitiated [17]. Fifty‐nine percent of patients had persistently high platelet levels even when chemotherapy was restarted, and 41% of patients did not have a recurrence of thrombocytopenia for the full length of their survival [17]. Tables 1 and 2 summarize the technique and outcomes of PSEs performed on cancer patients in the literature.

**TABLE 1:** Review of the literature regarding cancer patients that had a PSE (cohort studies).

Study	Study design	Demographic details	Platelet count pre‐PSE	Target platelet count and target outcomes	PSE procedure	Repeat PSE?	Outcome: efficacy and duration in improving platelet count	Outcome: did the patient end up receiving chemotherapy/surgery?	Length of hospitalization	Complications
Luz et al., 2016	Prospective phase II study.	33 GI cancer patients with chemotherapy‐induced thrombocytopenia.	Mean: 69 × 10^9^/L	Target 1: Thrombocyte level > 130 × 10^9^/L Target 2: Return to chemotherapy	‐ Lower splenic pole embolized ‐ Embolic agent: 1 vial of 100–300 microspheres. ‐ Infarcted 50%–70% of spleen.	Repeated in 2 patients.	‐ 94% of patients reached target platelet count (86% reached it 2 weeks after PSE). ‐ Mean platelet counts at Weeks 1–4 post‐PSE: 141 × 10^9^/L, 188 × 10^9^/L, 154 × 10^9^/L, 122 × 10^9^/L, respectively (decrease Week 3–4 as patients returned to chemotherapy)	All patients that needed to reinitiate chemotherapy treatment did so with a mean time to treatment return of 14 days.	‐ Patients′ immediate recuperation after PSE took 6 h ‐ When possible, same‐day discharge, but if symptomatic, patients stayed overnight.	‐ No adverse effects of Grade 3 or above in patients. ‐ The worst complication was overnight GI bleeding post‐PSE (without hemodynamic instability). Patient was discharged within 24 h. ‐ 12 patients had referred low‐to‐moderate pain, 3 patients had severe pain, 7 patients had nausea, and 7 patients had low‐grade fever
Kauffman et al., 2008	Retrospective review	28 patients with GI cancer at the time of PSE. Thrombocytopenia due to hypersplenism and splenomegaly.	81 × 10^9^/L	Primary target: platelet count increase greater than 150 × 10^9^/L Secondary target: starting systemic therapy	‐ Embolic agent: either gelatin sponge material or particulate agents. ‐ Percent of parenchymal occlusion: mean of 65%.	‐ Two patients repeated PSE.	‐ When procedures had sufficient follow‐up, the primary target (platelet count) was achieved in 96.3% of patients (26/27). ‐ Platelet count peaked at 293 × 10^9^/L post‐PSE. ‐For 23 patients that repeatedly had laboratory follow‐up, the average time to a platelet count above target (> 150 × 10^9^/L) was 10 days.	‐ Systemic therapy was started in 95.7% of patients (22/23). ‐ Average time to start systemic therapy was 32 days post‐PSE. After PSE, patients that underwent systemic therapy received an average of 4.5 cycles. 6 patients had a recurrence of thrombocytopenia that interfered with systemic therapy. ‐ Mean survival was 9.4 months post‐PSE.	Mean hospital stay was 4.5 days.	1 patient had an extended hospital stay (23 days) as a result of developing ileus, ascites, and then right upper lobe pneumonia. 1 patient was readmitted within 30 days of PSE for left‐sided pneumonia. ‐ All patients had post‐PSE abdominal pain. ‐ Fever in 16 patients. ‐ Pulmonary consolidation/atelectasis or effusion in 10 patients. ‐ 2 patients developed inguinal hematomas that were successfully treated.
Hidaka et al., 2009	Prospective study	20 HCC patients with thrombocytopenia.	Patients with marked thrombocytopenia: < 50 × 10^9^/L Patients with mild thrombocytopenia (< 80 × 10^9^/L) + decreased prothrombin activity.	Primary target: increase in platelet count 2 weeks after the last PSE greater than 50 × 10^9^/L in cases of marked thrombocytopenia. Increase in prothrombin activity in cases of mild thrombocytopenia Secondary target: start radiofrequency ablation (platelet count of > 50 × 10^9^/L is recommended to start ablation therapy)	‐ Super‐selective catheterization of the splenic lower or middle artery was done. ‐ Embolization by injecting gelatin sponge split into 2–4 mm cubes (suspended in saline). ‐ Splenic infarction ratio of 50%–70% achieved. ‐ Two days post‐PSE, CT volumetry was used to determine the infarction rate. If infarction was found to be < 50% after the first PSE, a second PSE was done 3 weeks later.	Treated once or twice with PSE. Mean number of PSE procedures was 1.4.	‐ PSE was successful in 19/20 patients (one patient dropped out of the study) ‐ Two weeks post final PSE, platelet count improved significantly—(38 ± 14 × 10^9^/L vs. 97 ± 43 × 10^9^/L), and prothrombin activity increased significantly (59.3 ± 19.8% vs. 65.2 ± 17.9%)	18/19 patients started radiofrequency ablation. Some patients that had a PSE could get additional therapies due to the ongoing increase in platelet count post‐PSE.		‐ No major complications during PSE. ‐ Post‐PSE, 12 patients had left‐sided abdominal pain (60%), 9 patients had fever > 38.5 °C (45%) and 1 patient had a left‐sided pulmonary effusion.
Jacome et al., 2020	Retrospective study	110 gastrointestinal cancer patients with thrombocytopenia due to hypersplenism. Most patients had colorectal cancer.	Median platelet count pre‐PSE: 77 × 10^9^/L	Target platelet count: greater than 100 × 10^9^/L	Median infarction rate of vascularization of the spleen was 70%.	6 patients had a repeat PSE. The second PSE was usually less efficacious than the first PSE.	‐ Median time to recover the platelet count post‐PSE was 28 days ‐ Post‐PSE 80% of patients had a platelet count greater than or equal to 100 × 10^9^/L ‐ Median platelet count post‐PSE: 175 × 10^9^/L ‐ Percent of patients who had ≥ 100 × 10^9^/L (80%), ≥ 130 × 10^9^/L (67%), and ≥ 150 × 10^9^/L (62%) post‐PSE ‐ Bevacizumab does not affect the efficacy of PSE for patients with chemotherapy‐induced hypersplenism.	‐Post‐PSE systemic therapy was continued in 81% of patients.	Median hospitalization was 2 days.	Most common complications post‐PSE: 83% had pain, 22% had fever, and 7% had pneumonia.
Passhak et al., 2018	Retrospective review	10 cancer patients (9 had metastatic colorectal cancer, 1 pancreatic cancer) with thrombocytopenia likely due to hypersplenism	Mean: 64.5 × 10^9^/L		Catheter tip was proximal to the main splenic artery. This artery was embolized by injecting particles (300–700 nm) such that a 50% reduction in splenic parenchyma blush was reached.	One patient had a repeat PSE.	Mean platelet count within 10–14 days post‐PSE: 224 × 10^9^/L 1 patient had a platelet count less than 100 × 10^9^/L 2 weeks after the procedure.	‐ Chemotherapy was reinitiated a mean of 18 days post PSE in 9 patients. ‐ 1 patient had a local treatment of his liver metastasis administered (not chemotherapy). ‐ Prolonged thrombocytopenia occurred again in 4 patients, one of whom had a repeat PSE as a result. ‐ Mean duration of ability of patients to undergo chemotherapy post‐PSE was 13.5 months ‐ Median time of chemotherapy post‐PSE was 10 months.	Mean hospitalization was 2.5 days (range 2–5 days).	Post‐PSE: All patients had mild abdominal pain for a few days. One patient had post‐embolization syndrome (fever and pain).
Bhatia et al., 2015	Retrospective review	13 patients that had an underlying malignancy (pancreatic adenocarcinoma, cholangiocarcinoma, or other) and chemotherapy‐induced thrombocytopenia.	88 × 10^9^/L		Proximal splenic artery was embolized using platinum coils or detachable Interlock coils.		‐ PSE successful in all patients ‐ After PSE: peak platelet count was 209 × 10^9^/L at a mean short‐term follow‐up of 35 days ‐ At mean follow‐up time of 9.2 months post‐PSE, platelet count was 152 × 10^9^ (mean) ‐ Mean splenic infarct rate was 29.5% at 0.5–12‐month follow‐up	‐ All patients were able to initiate chemotherapy. ‐ The baseline mean platelet count before starting chemotherapy was 162 × 10^9^ ‐ Time to start chemotherapy post‐PSE was a mean of 22 days in 12 patients. 1 patient refused chemotherapy. ‐ Patients were administered a mean of eight cycles of chemotherapy after PSE. One patient had thrombocytopenia that prevented them from completing the first cycle of chemotherapy.	The majority of patients were discharged 4–6 h after the procedure; one patient was admitted for 23 h for pain control.	Some patients had no adverse events, some had abdominal pain, and one patient had post‐embolization syndrome (low‐grade fever and left abdominal pain that radiated to the left shoulder) No major adverse effects in any patient.
Hill et al., 2020	Retrospective review	98 cancer patients (gastrointestinal malignancy was the most common type of cancer, and colorectal cancer was the most common diagnosis)	< 100 × 10^9^/L was considered thrombocytopenia in cancer patients.	Primary outcome: identify what predicts outcomes (i.e., platelet response) post‐PSE. Secondary outcome: whether patients who had PSE to reinitiate chemotherapy could do so. Also looked at splenic volume over time to see how long the embolization was effective for.	‐ Embolization using proximal or distal technique ‐ Gelatin sponge slurry combined with 80 mg gentamycin was used for embolization (this was done for most patients). ‐ 50%–75% of splenic parenchyma was embolized.	3/98 had a repeat procedure (results of the repeat were censored).	Patients were complete responders (platelets never went below 100 × 10^9^/L after PSE), partial responders (first platelets increased, then they decreased and went below 100 × 10^9^/L), and nonresponders (platelets never went above 100 × 10^9^ post‐PSE) ‐ 58 (59%) were complete responders ‐ 28 (29%) were partial responders ‐ 12 (12%) patients had no response For CR patients, there were persistently high platelet counts even when chemotherapy was restarted. The persistent elevation in platelet count was linked with a long‐lasting decrease in spleen volume.	‐ Of the patients who had PSE to restart chemotherapy, 97% of patients started at least one line of chemotherapy after PSE. Median time to reinitiate chemo post‐PSE: 25 days ‐ Of the patients that had PSE to get a surgery, all of them had surgery. ‐ 41% of patients did not have a recurrence of thrombocytopenia for the rest of their lifetime.		2% of patients died from complications linked to PSE. One patient had a bacteremia 4 days after PSE, which resolved with antibiotics—during hospitalization the patient chose to go to hospice care and later died. Another patient had left lower lobe pneumonia and sepsis 7 days after PSE. Condition became worse, multiorgan failure, went for hospice care, died. Major complication rate was 8%—persistent post‐embolization syndrome symptoms leading to readmission was the most common major complication. Minor complications: pneumonia (4 patients) and groin site pruritus (1 patient).
N′Kontchou et al., 2005	Retrospective review	32 cirrhotic patients; 8 of these had HCC—these were the 8 whose data were included in the table	34–59 × 10^9^/L	Likely platelet count over 70 × 10^9^/L to be able to have percutaneous ablation of HCC.	‐ Tip of catheter inserted in splenic hilum or in intrasplenic arterial branches (as distally as possible) ‐ Embolization particles: polyvinyl alcohol foam or microspheres that were 200–1000 μm ‐ Embolization was done by repeatedly injecting particles until blood flow through the spleen decreased to 50%. ‐ 30%–70% necrosis was achieved.	No	Platelet count range: 76–140 × 10^9^/L	Percutaneous ablation of HCC was done in 7/8 patients, with the tumor being completely necrosed.		2/8 patients had severe complications: 1 had portal vein thrombosis, and the other had bleeding of the puncture site. None died specifically because of PSE.
Tomikawa et al., 2010	Observational, retrospective	43 cirrhotic patients—18 of which had the laporascopic splenectomy or PSE for anticancer treatment of HCC—the data from these 18 were included in the table ‐ 4 underwent laporoscopic splenectomy ‐ 14 underwent PSE			‐ Catheter tip was advanced as distally as possible within an artery within the spleen. ‐ Gelatin sponge used for embolization. ‐ Planned amount of devascularized parenchyma was 60%–70% (determined by parenchymal phase angiography)		Platelet count at start of anticancer therapy: ‐ Laporascopic‐spelenctomy group: 240 ± 55 × 10^9^/L ‐ PSE group: 98 ± 40 × 10^9^/L	All the patients received anticancer treatment (in the cases of either intervention). Anticancer therapies encompass ablation, hepatectomy, transarterial chemoembolizationa, and intra‐arterial chemotherapy).		
Loffroy et al., 2019	Retrospective single‐center observational study	8 cancer patients with malignancies from the GI tract.	Mean: 74 × 10^9^/L	Primary goal: platelet increase > 150 × 10^9^/L Secondary goal: initiating systemic chemotherapy.	‐ Branches that supply blood to the lower pole of the spleen were embolized with a Glubran®2/Lipiodol® mixture (distal embolization). ‐ Mean splenic infarction post‐PSE was 55% (based on CT, average of 19 days post‐PSE)	No	PSE had a technical success of 100%. The primary and secondary goals were achieved in 100% of patients (that had adequate follow‐up). For the 7 patients that received laboratory follow‐up: ‐ Mean platelet count rose to a peak of 272 × 10^9/L 10 days post‐PSE. ‐ Mean platelet count 30 days after PSE: 195 × 10^9^/L	Post‐PSE, the 7 patients that had adequate follow‐up initiated systemic chemotherapy within 30 days.	Mean hospital discharge was 3.3 days post‐PSE.	No major complications. 5 patients had moderate and temporary post‐embolization syndrome (abdominal pain, nausea, and low‐grade fever); 4 patients experienced referred low‐to‐moderate abdominal pain; 3 had nausea; and 3 had low‐grade fever.
Kis et al., 2020	Retrospective study	35 thrombocytopenic cancer patients with hypersplenism/splenomegaly due to chemotherapy, portal hypertension, or hematologic malignancy.	Mean: 65.7 ± 19.7 × 10^9^/L	Success: platelet count above 100 × 10^9^/L OR symptoms that led to the PSE being performed are resolved.	‐ 300–500 μM tris‐acryl gelatin microspheres used for embolization. ‐ Main target of embolization was the inferior splenic segments. ‐ 50%–60% reduction in splenic parenchymal function was reached. ‐ After the first 11 PSE procedures were performed, NSAIDs, corticosteroids, and celiac plexus neurolysis were incorporated into the postprocedural management protocol January in 2015.	4 patients had repeat PSEs.	‐ The platelet count rose to a peak value of 221 ± 83 × 10^9^/L (2 weeks post‐PSE). ‐ The most current platelet count of patients with more than a year‐long follow‐up was 174 ± 113 × 10^9^/L ‐ The increase in platelet count was significantly higher in the chemotherapy‐induced group compared to the portal hypertension cause group (even after a lengthy follow‐up period) ‐ PSE resulted in a 59 ± 16% splenic infarct size in patients.	Technical success of PSE: 100%. Overall clinical success rate: 86%—5 patients did not have a significant increase in platelet count after their PSE. Clinical success rate for patients with: ‐ chemotherapy‐induced splenomegaly: 88% ‐ portal hypertension‐induced splenomegaly: 90% ‐ hematologic malignancies inducing splenomegaly: 75%. For patients where the main indication for patients to receive a PSE was thrombocytopenia, 84% (26/31) of them could initiate cancer treatments (24 had chemotherapy, 1 had percutaneous ablation). The main indication for PSE was GI bleed for 3 patients and ascites for 1 patient; the PSE resolved these issues.	Overall average hospital stay: 2.6 ± 3.7 days (hospital stay was longer before January 2015, shorter after)	44% of PSEs had major complications (not defined): ‐ Rate for hematological malignancy group: 64% ‐ Rate for portal hypertension‐induced group: 30% ‐ Rate for chemotherapy‐induced group: 39% 54% of PSEs had minor complications. Most common complication: abdominal pain: 9 instances of severe pain, 7 of moderate, 20 of mild. 9 patients: Grade 1 fever 3 patients: Grade 1/2 pleural effusion and/or ascites, 4 patients: Grade 3 pleural effusion and/or ascites 2 patients: splenic subcapsular hematoma 2 patients: asymptomatic non‐occlusive thrombosis Other: Left lower lobe pneumonia, DVT, pancreatitis The inclusion of NSAIDs, corticosteroids, and celiac plexus neurolysis in post‐PSE management lowered the rate of major complications from 73% to 46%. Also lowered the rate of moderate or severe pain from 92% to 20%.

**TABLE 2:** Review of the literature regarding cancer patients that had a PSE (case reports and case series).

Study	Study design	Demographic details	Platelet count pre‐PSE	Target platelet count and target outcomes	PSE procedure	Repeat PSE?	Outcome: efficacy and duration in improving platelet count	Outcome: did the patient end up receiving chemotherapy/surgery?	Length of hospitalization	Complications
Rainusso et al., 2021	Case report	6 y/o patient with metastatic desmoplastic small round cell tumor and hypersplenism‐related thrombocytopenia.	< 30 × 10^9^/L	Return to chemotherapy	‐ Lower pole branches of the distal splenic artery were embolized. ‐ Embolic agent: 500–700 μm size Embosphere. ‐ Decreased perfusion of about 50% of the caudal spleen.		Platelet count post‐PSE: increased to > 50 × 10^9^/L	‐ Patient received two more cycles of chemotherapy post‐PSE, then underwent splenectomy and exploratory laparotomy with complete resection of pelvic and intraabdominal tumors.		Fever, vomiting, small hematoma at insertion site of catheter

Nakatsubo et al., 2021	Case series	2 patients with gastric cancer and hypersplenism associated with gastric cancer with splenic vein invasion. Thrombocytopenia and splenomegaly.	Case 1: Prechemotherapy: 179 × 10^9^/L 2 months after starting chemotherapy: 58 × 10^9^/L Case 2: Prechemotherapy: 213 × 10^9^/L 2 months after starting chemotherapy: 55 × 10^9^/L	Return to chemotherapy	Case 1: ‐ Embolic agent: gelatin sponge fragments. ‐ 70% embolization attained. Case 2: ‐ Same as Case 1, but 60% embolization was achieved.		Case 1: 2 weeks post‐PSE platelet count increased to 310 × 10^9^/L. 6 months after PSE, no thrombocytopenia was present. Case 2: 2 weeks post‐PSE, platelet count increased to 230 × 10^9^/L. No thrombocytopenia was observed for about 4 months post‐PSE.	Chemotherapy was continued in both patients post‐PSE. Case 1: 2 weeks post‐PSE, chemotherapy treatment was resumed. Case 2: Ramucirumab–paclitaxel treatment was administered.	Case 1: Hospital stay 15 days. Case 2: Hospital stay 14 days	Case 1: No serious complications. Patient had a mild fever for 5 days post‐PSE that resolved. Case 2: No serious complications.

Beji et al., 2015	Case series	3 cases of thrombocytopenia in cancer patients due to hypersplenism.	Case 1: 80 × 10^9^/L Case 2: 60 × 10^9^/L Case 3: 80 × 10^9^/L	Case 1: Target–120 × 10^9^/L Case 3: Target–120 × 10^9^/L	Case 1: ‐ Branches of the splenic artery at the inferior pole and middle third of the spleen were embolized. ‐ Embolic agent: Uncharged 250‐micron microspheres. ‐ Seven days post‐PSE, CT indicated 50%–70% of the spleen was ischemic. Case 2: Same method as Case 1. 50%–70% splenic ischemia. Case 3: Same method as Case 1. 70% splenic ischemia.		PSE resulted in platelet count > 150 × 10^9^/L Case 1: 10 days post‐PSE platelet count was 162 × 10^9^/L Case 2: One month post‐PSE platelet count was 200 × 10^9^/L ‐ At 4 months post‐PSE, platelet count was stable at 120 × 10^9^/L Case 3: 15 days post‐PSE, platelet count was stable at 530 × 10^9^/L	Chemotherapy was resumed or started. Case 1: 10 days post‐PSE systemic therapy could be initiated. Case 2: One month post‐PSE patient could start clinical trial with angiogenics. Case 3: 15 days post‐PSE, antiangiogenic therapy was initiated again, and the patient was added to a new therapeutic trial.	Case 1: Hospitalized for 48 h post‐PSE to treat post‐embolization syndrome.	Case 1: Had post‐embolization syndrome. Left upper quadrant pain for 48 h post‐embolization. Case 2: No complications at time of embolization. At 4 months post‐PSE, secondary pulmonary and vertebral lesions formed, leading to pain that did not respond to analgesics. Case 3: Post‐PSE patient had pain resistant to morphine‐based analgesics. Hyperthermia for 48 h.

Heianna et al., 2016	Case report	28 y/o woman with advanced sigmoid colon cancer and multiple liver metastases. Had splenomegaly and thrombocytopenia that were caused by portal hypertension due to oxaliplatin administration.	54 × 10^9^/L		‐ Performed selective angiography of the major branches of the splenic artery using a microcatheter. ‐ 40%–50% splenic embolization (termed “mild” PSE) ‐ During PSE, contrast medium mixed with 0.5 g of cefazolin and microcoils was employed to verify the embolus range.		‐ Day 7 after mild PSE: platelet count was 121 × 10^9^/L ‐ Two months post mild PSE, reduction in splenic volume observed. Platelet count of 90–170 × 10^9^/L maintained post mild PSE.	‐ Day 7 post‐PSE chemotherapy was resumed. ‐ Patient continued chemotherapy without discontinuation for about 2 years after the mild PSE until her passing due to liver failure.		Pain that was controlled with analgesic.

Dinkelaar et al., 2011	Case report	63 y/o patient with splenomegaly caused by thrombocytopenia as a result of mantle cell lymphoma (stage IVa). Received high‐dose chemotherapy for mantle cell lymphoma. Has CHD and nonsmall cell lung carcinoma.			‐ Angiography was performed (via femoral artery), which showed patent splenic artery coming from the celiac trunk. ‐ Selective catheterization of the splenic artery just distal to the dorsal pancreatic artery origin. This was embolized with multiple platinum coils. ‐ Embolization was successful, and the dorsal pancreatic artery retained patency.					‐ A few hours post‐PSE, patient felt abdominal pain in the left upper quadrant ‐ Mild abdominal pain on compression in that region, with no signs of peritonitis ‐ He had high blood pressure (170/115) ‐ The patient was discharged 2 days later (with mild abdominal pain). ‐ At home, the patient experienced worsening abdominal pain and weakness. ‐ 3 days post‐PSE, patient collapsed and was admitted to ER. Pain in the left upper quadrant (no signs of peritonitis). Patient had hemodynamic shock with renal failure, metabolic acidosis, severe necrosis, and elevated infection values. ‐ It is believed patient experienced severe sepsis or tumor lysis syndrome because of the large splenic infarction post‐PSE. Patient died in the span of a few hours. ‐ Autopsy: severe ischemia of colon and small intestine. Very heavy spleen that was completely avascular (and had coils in the splenic artery due to PSE). Adenocarcinoma and pheochromocytoma were found.

Kaneko et al., 2019	Case report	84 y/o woman with advanced gastric cancer with bleeding and immune thrombocytopenic purpura.	< 50 × 10^9^/L	Target 1: platelet count above 50 × 10^9^/L Target 2: to have combined subtotal gastrectomy and splenectomy	‐ PSE was performed by transcatheter embolization of the inferior branch of the splenic artery. ‐ After PSE, the patient received high dose immunoglobulin.		Increased platelet count due to PSE and high‐dose immunoglobulin therapy: 56 × 10^9^/L on day of gastric resection	‐ Patient had combined subtotal gastrectomy and splenectomy 14 days post‐PSE. ‐ Postoperative adjuvant chemotherapy was not administered due to the patient’s old age. No recurrence of cancer at 32 months postsurgery (and platelet count was normal)		

Shimizu et al., 2003	Case report	67 y/o man diagnosed with HCC and thrombocytopenia due to liver cirrhosis.	30 × 10^9^/L		‐ Arterial catheter in splenic hilum, injection of 2 by 2 mm squares of antibiotic‐impregnated Gelfoam into the splenic parenchyma. ‐ Splenic volume decreased to about 50%.		2 months post‐PSE platelet count increased to: 60 × 10^9^/L.	2 months after PSE, hand‐assisted laparoscopic partial hepatectomy was done.	Discharge 12 days posthepatectomy.	No serious complications.

Mizukami et al., 2023	Case report	43 y/o Rectal cancer patient. After the sixth course of systemic therapy, splenomegaly was exacerbated and stomal varices formed. 5 months after the stomal varices formed, patient had a percutaneous transhepatic stoma variceal embolization (PTO). 5 months after the PTO, he had a PSE to improve the splenomegaly and thrombocytopenia.	52 × 10^9^/L at time of PSE.		‐ Embolization of middle and inferior pole branches of splenic artery ‐ Used Spongel (c) for embolization ‐ 80% embolization effect.		‐ Platelet count improved: approximately a few weeks after the procedure, platelet count was 256 × 10^9^/L ‐ A little over 2 months after the procedure: platelet count slightly decreased to roughly about 180 × 10^9^/L	‐ Returned to chemotherapy after PSE ‐ 5 months post‐PSE, no stomal bleeding, and the thrombocytopenia has been alleviated.		Not stated.

Kogure et al., 2016	Case report	HCC patient with portal hypertension and splenomegaly. PSE is done to lower portal pressure.	57 × 10^9^/L		Catheter was inserted in the splenic artery. Embolization with 2 mm‐square gelatin sponge.		7 days after PSE, platelet count increased to 153 × 10^9^/L 1 year after PSE: 81 × 10^9^/L 5 years after PSE: 82 × 10^9^/L 5 years post‐PSE: CT volumetry showed splenic volume was within 40% of the original volume.	Follow‐up after 13 years: there were no varices or variceal bleeding (until the patient passed away due to his cancer).		Post‐PSE, patient developed mild left upper abdominal pain and low‐grade fever.

Pinto et al., 2005	Case report	30 y/o man with cirrhosis and hypersplenism with splenomegaly had a recurrence of craniopharyngioma.	40 × 10^9^/L		‐ Spleen was embolized using 250–355 μm polyvinyl alcohol particles. ‐ Embolization of 60% of splenic parenchyma.		Within 1 week, the platelet count increased. The improvement in platelet count continued for 85 days post‐PSE.	The patient got a stereotactic injection of radioactive P32 and did not have bleeding complications (due to improved platelet count).	Discharged 4 days post‐PSE (but later readmitted)	Mild post‐embolization syndrome: fever (2–3 days after PSE) Patient readmitted for fever and chills that occurred as a result of pneumonia (treated with antibiotics)

Saddekni et al., 2016	Case report	77 y/o patient with chronic lymphocytic leukemia (CLL) and hypersplenism (associated with the CLL). Had pancytopenia.	67 × 10^9^/L		Authors were first unsuccessful at catheterizing the splenic artery by route of the celiac trunk as a result of substantial atherosclerotic disease. Through imaging, it was revealed that the right gastroepiploic artery communicated with the distal splenic artery via its anastomosis with the left gastroepiploic artery. This anastomosis was patent. Catherizing the distal splenic artery by moving the catheter from the gastroduodenal artery through the gastroepiploic arteries was possible and was done. Lower 2/3 of spleen was embolized by 500 μm polyvinyl alcohol particles. 60%–70% reduction in spleen size on follow‐up.		Platelet count increased to 333 × 10^9^/L 2 years post‐PSE: 53% decrease in spleen volume		Discharged 4 days post‐PSE	Left upper abdominal pain (scored 8/10)

Gonsalves et al., 2012	Case series	3 cancer patients (1 rectal cancer (61 y/o), 2 HCC (46 y/o, 64 y/o)).	‐ Rectal cancer 61 y/o: 66 × 10^9^/L ‐ HCC 46 y/o: 58 × 10^9^/L ‐ HCC 64 y/o: 42 × 10^9^/L		‐ For 2 patients, their splenic artery split into superior and inferior terminal branches that then split further within the spleen. One of these terminal branches was slowly embolized with the Onyx nonadhesive liquid embolization system. ‐ For 1 patient, two splenic arterial branches were embolized due to complex anatomy. ‐ Splenic parenchymal embolization was 40%–60%.	No	Platelet count 2 weeks post PSE: ‐ Rectal cancer 61 y/o: 451 × 10^9^/L ‐ HCC 46 y/o: 112 × 10^9^/L ‐ HCC 64 y/o: 77 × 10^9^/L Platelet count in longest follow‐up period: ‐ Rectal cancer 61 y/o: 313 × 10^9/L (9 months post‐PSE) ‐ HCC 46 y/o: Died as a result of tumor progression 38 days post‐PSE ‐ HCC 64 y/o: 191 × 10^9^/L (6 months post‐PSE)	‐ PSE was successful in all the patients. ‐ Post‐PSE, splenic infarction percentage was 77% in the rectal cancer patient (1 month post‐PSE), 51% in 46 y/o HCC patient (3 months post‐PSE) and 32% in 64 y/o HCC patient (1 month post‐PSE). ‐ Thrombocytopenia fixed in all patients. ‐ Chemotherapy started on Day 38, Day 18, and Day 60 (for rectal cancer, 46 y/o HCC and 64 y/o HCC patients, respectively).	All patients were discharged 1 day after PSE.	No complications.

Sato et al., 2019	Case series (3 patients—only one patient had a PSE alone with no additional procedures; their data were included in the table)	HCC patient who developed Grade 3 thrombocytopenia caused by the lenvatinib therapy (previously treated with transarterial chemoembolization).	About 40 × 10^9^/L				Post‐PSE platelet count: 140 × 10^9^/L	Post‐PSE, patient continued to receive lenvatinib therapy for over 6 months.		No serious complications

In Case 1, the patient underwent a successful PSE with a platelet count exceeding the desired target for treatment (75 × 10^9^/L) a few days after the procedure. However, this platelet count lasted for 18 months. Zhu et al. found that in order to achieve long‐term improvements in platelet count, interventional radiologists should aim for a minimum of 50% splenic infarction [20]. They found that in cirrhotic patients with hypersplenism, those that had a splenic infarction percentage of 50% or more continued to have a significantly higher platelet count than their preprocedure values from the second week to 5 years post‐PSE [20]. Yet, patients with < 50% splenic infarction only experienced significant improvements in platelet count for 6 months [20]. Our patient’s percent embolization was 30%–35%, which could potentially explain recurrent thrombocytopenia, in addition to other factors such as chemotherapy dose increase. The percent embolization may have been limited, as the patient’s splenomegaly could have led to a high infarction volume, which is a risk factor for complications [21]. It is also important to note that the degree and longevity of platelet elevation post‐PSE is multifactorial. Cai et al. found that platelet increment post‐PSE in cirrhotic patients is significantly associated with cholinesterase levels, noninfarcted splenic volume as well as splenic infarction ratio [21].

In Case 2, the intention was to infarct only part of the spleen. However, the whole spleen, in addition to the tail of the pancreas, was inadvertently infarcted, resulting in catastrophic consequences such as necrotizing pancreatitis. The splenic artery branches into multiple components, including the dorsal and greater pancreatic arteries, that supply the body and tail of the pancreas, respectively [22]. As a result, there is a risk of inadvertently embolizing these pancreatic arteries during a PSE [22–28]. This is a rare complication according to our literature review. Infarction of the whole spleen is associated with a high incidence of splenic abscess due to impairment of the spleen’s immune function [29]. In order to minimize the complications of a PSE, the splenic embolization or infarction rate was an important factor considered in numerous cancer studies, often aiming for 50%–75% [9, 11, 15, 17, 30]. A systematic review by Talwar et al. found that in a series of studies that reported infarction rates and complications post‐PSE, 45% of patients that had a greater than 70% splenic infarction had major complications, while only 5% of patients that had a splenic infarction lower than or equal to 70% faced major complications [31]. Additionally, the middle and/or inferior pole of the spleen was often embolized in cancer patients, likely to reduce the risk of subdiaphragmatic abscess or pleural effusion [6, 11, 30].

Understanding how to prevent nontarget embolization is crucial to minimize complications. Estimating the splenic volume that has been infarcted intraprocedurally is a difficult task, and most methods strongly rely on operator experience [32, 33]. The percent of splenic volume that a specific arterial branch supplies cannot be accurately quantified, and this difficulty in quantification may lead to the rare complication of total (instead of partial) splenic embolization [33]. Usually, contrast‐enhanced CT is done a couple of weeks post‐PSE to precisely estimate the infarcted spleen volume [32]. Being able to use imaging intraprocedurally to estimate the relative amount of ischemic splenic tissue present has the potential to reduce the likelihood of excessive embolization and associated complications [32]. Ishikawa et al. demonstrated that the splenic infarction rate can be measured using cone beam CT angiography during the procedure [34]. Cone beam CT provides three‐dimensional information about soft tissue and vascular anatomy, allowing interventionalists to receive intraprocedural feedback on infarction rate that may help avoid excessive embolization [34, 35]. Moreover, Ou et al. used a method that took into account the diameters of the branches of the splenic artery to estimate the embolization ratio intraprocedurally [33]. These diameters were measured using 2D angiographic images during the procedure [33]. Lu et al. also posited the idea of using a specific embolic material (8 spheres), wherein each vial corresponds to a certain splenic embolic volume, to control the degree of embolization during the PSE [36].

Acquiring a deeper understanding of the patient’s anatomy could help guide a safer embolization. Although there are few communicating branches between the various segments of the spleen, some variations exist in patients such that they have intra‐ and extra‐splenic anastomosis (15.3%–43.3% and 6.6%–15.3% of people, respectively) as well as certain variations in splenic artery anatomy [7]. This could complicate a PSE and its associated outcomes [7].

Overall, these cases demonstrate that although a PSE can benefit a cancer patient, there can be grave complications associated with the procedure when inadvertent total and nontarget embolization occurs. A key guiding principle that can be drawn from both cases is that while a low percent embolization may lead to a less durable rise in platelet count, it is better to embolize in a staged fashion, as opposed to risking excessive embolization in a single session, which could lead to severe complications. Understanding how to enhance the effectiveness and safety of PSEs in cancer patients is essential to achieve successful and durable outcomes. More research should be done to determine how the risk of nontarget/excessive embolization can be reduced during a PSE.

## Author Contributions

Zahra Ridha and Mohammad Refaei conceptualized the project. Zahra Ridha reviewed the patient data, compiled it, and wrote the entirety of the manuscript. Zahra Ridha conducted the literature search and review. Kiat Tsong Tan, Michael Connolly, Radhika Yelamanchili, Rachel VanderMeer, and Mohammad Refaei reviewed the manuscript and edited it, providing their expertise to improve the text.

## Funding

No funding was received for this project.

## Disclosure

All authors have read and approved the final version of the manuscript. Zahra Ridha had full access to all of the data in this study and takes complete responsibility for the integrity of the data and the accuracy of the data analysis.

## Ethics Statement

Informed consent was obtained through the approved means of the hospital’s ethics guidelines.

## Consent

The two patients provided informed consent for the publication of their anonymized clinical information.

## Conflicts of Interest

The authors declare no conflicts of interest.

## Supporting Information

This case report abided by the CARE checklist of case report guidelines.

## Supporting information


**Supporting Information** Additional supporting information can be found online in the Supporting Information section.

## Data Availability

The data that support the findings of this study are available on request from the corresponding author. The data are not publicly available due to privacy or ethical restrictions.
