# Supplementary figures and images for: Splenic Embolization to Manage Thrombocytopenia in Cancer Patients: Case Reports and Review of the Literature
Source: Case Rep Hematol. 2026 Feb 13;2026:3779663. doi: 10.1155/crh/3779663 (PMC12905002; doi:10.1155/crh/3779663)

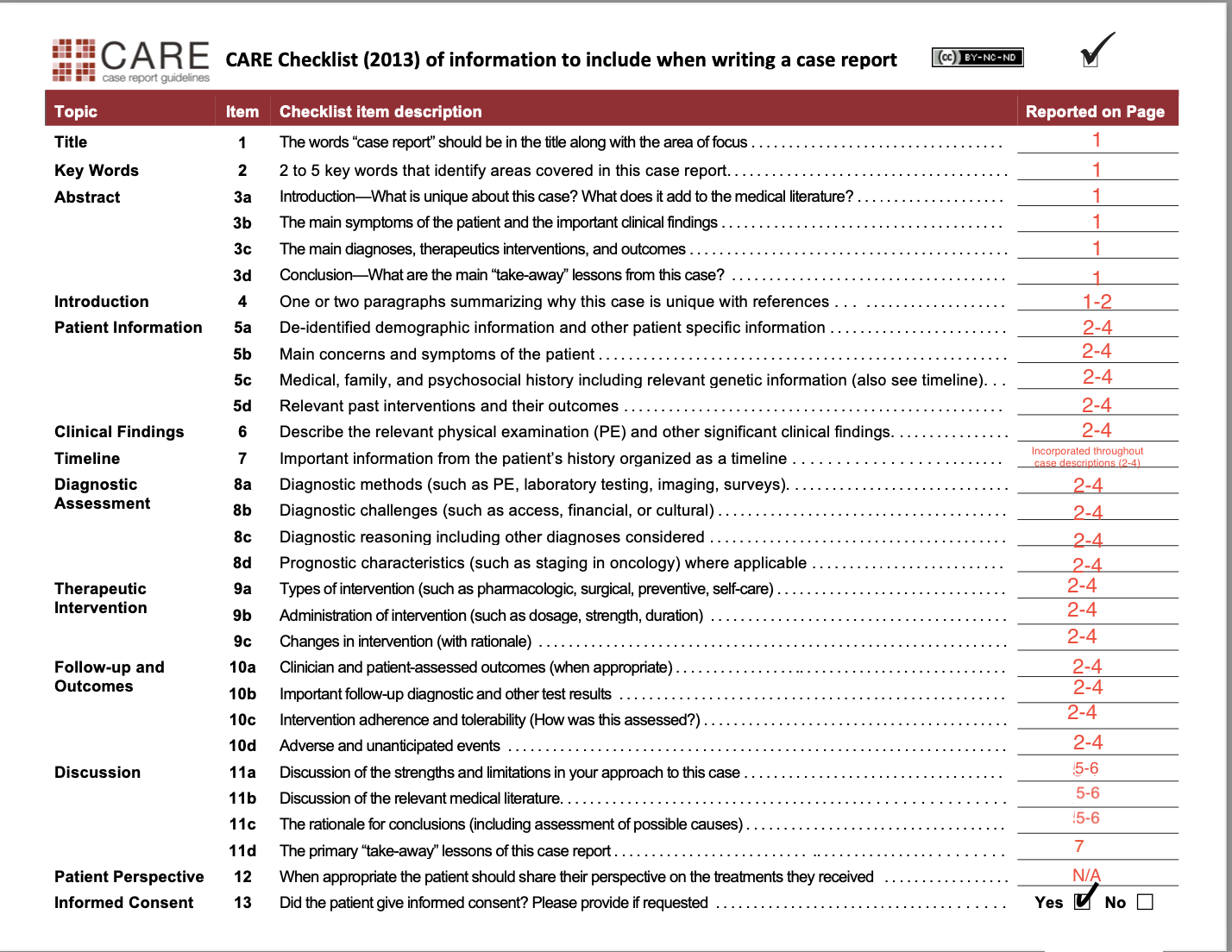

Supplement: Supplementary file 1 — Supporting Information Additional supporting information can be found online in the Supporting Information section. [file CRH-2026-3779663-s001.png]
